# Cytotoxicity and concentration of silver ions released from dressings in the treatment of infected wounds: a systematic review

**DOI:** 10.3389/fpubh.2024.1331753

**Published:** 2024-02-21

**Authors:** Javier Sánchez-Gálvez, Santiago Martínez-Isasi, Juan Gómez-Salgado, José María Rumbo-Prieto, María Sobrido-Prieto, Miriam Sánchez-Hernández, María García-Martínez, Daniel Fernández-García

**Affiliations:** ^1^Doctoral Programme in Health, Disability, Dependence, and Welfare, University of León, León, Spain; ^2^Faculty of Nursing, Catholic University of Murcia (UCAM), Cartagena, Murcia, Spain; ^3^Simulation, Life Support, and Intensive Care Research Unit (SICRUS), Health Research Institute of Santiago de Compostela (IDIS), Santiago de Compostela, Galicia, Spain; ^4^Primary Care Interventions to Prevent Maternal and Child Chronic Diseases of Perinatal and Developmental Origin (RICORS) (RD21/0012/0025), Carlos III Health Institute, Madrid, Spain; ^5^CLINURSID Research Group, Department of Psychiatry, Radiology, Public Health, Nursing, and Medicine, University of Santiago de Compostela, Santiago de Compostela, Galicia, Spain; ^6^Department of Sociology, Social Work, and Public Health, Faculty of Labour Sciences, University of Huelva, Huelva, Spain; ^7^Escuela de Posgrado, Universidad de Especialidades Espíritu Santo, Guayaquil, Guayas, Ecuador; ^8^Department of Health Sciences, Faculty of Nursing and Podiatry of Ferrol, University of A Coruña, A Coruña, Spain; ^9^Knowledge Support Unit (USCO), Ferrol University Hospital Complex, Health District of Ferrol, Galician Health Service, Ferrol, Spain; ^10^Health Research Nursing Group (GREIS), Department of Nursing and Physiotherapy, University of León, León, Spain

**Keywords:** wounds and injuries, silver dressing, silver bandage, release experiment, ion-exchange, ion-liberation

## Abstract

**Introduction:**

Silver-releasing dressings are used in the treatment of infected wounds. Despite their widespread use, neither the amount of silver released nor the potential *in vivo* toxicity is known. The aim of this study was to evaluate the cytotoxic effects and the amount of silver released from commercially available dressings with infected wounds.

**Methods:**

The review was conducted according to the PRISMA statement. The Web of Science, PubMed, Embase, Scopus, and CINAHL databases were searched for studies from 2002 through December 2022. The criteria were as follows: population (human patients with infected wounds); intervention (commercial dressings with clinical silver authorized for use in humans); and outcomes (concentrations of silver ions released into tissues and plasma). Any study based on silver-free dressings, experimental dressings, or dressings not for clinical use in humans should be excluded. According to the type of study, systematic reviews, experimental, quasi-experimental, and observational studies in English, Spanish, or Portuguese were considered. The quality of the selected studies was assessed using the JBI critical appraisal tools. Studies that assessed at least 65% of the included items were included. Data were extracted independently by two reviewers.

**Results:**

740 articles were found and five were finally selected (all of them quasi-experimental). Heterogeneity was found in terms of study design, application of silver dressings, and methods of assessment, which limited the comparability between studies.

**Conclusion:**

*In vivo* comparative studies of clinical dressings for control of infection lack a standardized methodology that allows observation of all the variables of silver performance at local and systemic levels, as well as evaluation of its cytotoxicity. It cannot be concluded whether the assessed concentrations of released silver in commercial dressings for the topical treatment of infected wounds are cytotoxic to skin cells.

**Systematic review registration:**

https://www.crd.york.ac.uk/prospero/display_record.php?ID=CRD42022351041, PROSPERO [CRD42022351041].

## Introduction

Historically, silver has had multiple uses due to its bactericidal capacity (1). Dressings containing silver can control and reduce the bacterial load, and they cover a wide range of action, becoming an alternative to the use of local antibiotics. The silver must be released into the fluid and onto the wound surface. In order for silver to be released from the dressings, different silver salts and complexes are used, which dissolve when they come into contact with fluids (exudate moisture and application of hydrogels or water), thus releasing the silver ions (2). Silver ions, bearing a positive charge, display a robust affinity for negatively charged biomolecules like phosphorus and sulfur. These elements form the fundamental components of proteins, peptides, amino acids, and cell surface structures in both host and microbial cells. As a result, silver induces structural alterations in the cell wall and membrane of bacterial cells, leading to various morphological modifications (3). The movement of ions toward the bacterial cell is driven by the electrochemical gradient. In regions where the local electrochemical gradient attains a sufficient level, ions infiltrate the cell, disrupting the respiratory system and binding to Deoxyribonucleic Acid (DNA) (3).

These findings have led to the development of different types of silver-releasing dressings for local application prior to infection or in combination with antibiotic treatment: elemental silver, metallic silver (Ag), nanocrystalline particles, inorganic compounds/complexes (silver nitrate, silver sulphadiazine…), and organic complexes (colloidal silver formulations) (4). They all have different solubility, so they differ in their ability to deliver free silver ions. The amount of silver ions generated by these salts will depend on both their solubility and the dissolution medium (5).

Continued application in clinical practice varies between 48 h and 96 h (6), though it depends on different factors: type of dressing, amount of wound exudate and type of secondary dressing used (usually polyurethane foam and sodium acrylate), and dressing replacement while performing treatment. A period of 14 days is also recommended to observe the evolution of the treatment (7). There are a number of studies on silver release using animal models or done *in vitro*, but few *in vivo* studies on humans (8–13).

The methodology applied to simulate a wound varies across *in vitro* studies, both in the way the dressing is saturated (physiological saline, fetal bovine serum, water, buffer solution or phosphate buffer) (14–16), temperature (4 °C −37°C) (13, 17), the recipient device (specimens with different simulated wound fluid exudate, Franz diffusion cells, and bi-compartmental devices) (12–14), and the method of quantification (13, 17–19). The results obtained in these studies are poorly translatable to the clinical reality of applying a dressing to an infected wound. While these products are assumed to be safe and effective (18), scientific evidence on how they work (how they release silver, the amount released, the effectiveness of the dressing, or the possibility of transferring other dangerous metals besides silver to the wound bed) is based on inconclusive or even contradictory study methods (20).

It should be noted that the release of silver, beyond its bactericidal capacity, can produce adverse effects, such as toxicity effects of silver nanoparticles. *In vitro*, a level of 0.01 μg/ml of silver in an aqueous system may be sufficient to control bacteria. However, a higher concentration is likely to be required *in vivo* (21). The effective inhibitory concentration of silver nanoparticles varies between 10 μg/ml and 100 μg/ml (22). Factors influencing the cytotoxicity of silver nanoparticles encompass surface chemistry, crystallinity, shape, size, and capping agent. Additionally, environmental factors, such as ionic strength, pH, and the presence of complexing agents, also significantly contribute to determining their toxicity (23).

Prolonged utilization of silver dressings may result in the local and systemic absorption of silver, manifesting as transiently elevated levels detected in blood and urine, along with the deposition of silver in the lesion and various body organs. Despite the low incidence of elevated systemic Ag+ levels, implying a low risk of absorption from the normal application of a silver-releasing dressing and proper functioning of silver elimination pathways in the body, it is noteworthy that the human body in its normal state contains approximately 1 mg of silver. The smallest documented amount of silver capable of inducing generalized argyria in an individual ranges from 4 g−5 g to 20 g−40 g (24).

A preceding review suggests that, following application to healthy skin, a blood silver level of < 1 μg/l is considered normal. However, when applied through a wound, these levels exhibit variability, ranging from 38 μg/l−200 μg/l when silver sulphadiazine is applied, and 126 μg−200 μg of silver per liter when silver nanoparticles are used (13). Conversely, some authors do not posit the possibility of blood toxicity arising from the absorbed levels of silver (12). The lack of knowledge regarding the amount released and the relationship between the released amount and absorption motivates us to investigate the toxicity of silver in these products routinely applied in situations involving impaired skin integrity. Despite this knowledge, no safety profile is currently available, and the effect on keratinocytes and fibroblasts in the wound and perilesional skin is unknown. In topical applications of dressings, accumulation in the most superficial layers of the skin occurs rapidly (2 h−8 h), and within 28 days, no traces of silver are visible in the tissue. Yet, in prolonged treatments or with high concentrations, systemic absorption may occur (25). Hadrup et al. (13) argue that healthy skin is an effective barrier against silver, although it is weaker on mucosal surfaces. Therefore, in the event of loss of skin continuity, the barrier function would in turn be altered, thus increasing the permeability of silver (13). Some forms of silver can be absorbed into the bloodstream, with the possibility of settling in various organs (11). High amounts of silver accumulated in the dermis and epidermis may lead to a change in skin colouration or argyria, which, in the case of dressings, is self-limiting. Some of the absorbed silver reaches the bloodstream quickly, but only in the case of high silver ion levels can excretion in the urine be observed. The elimination of silver present in the bloodstream is slow, with a mean residual permanence of 46 days (25).

The only standardized protocol developed in Europe in this respect is prEN 17854 (26). This document evaluates the bactericidal and bacteriostatic capacity of the dressing, but not the concentration of silver or the cytotoxic effects on the patient. Additionally, none of the manufacturers of products with potentially toxic metals have described penetration capacity values, as these particles are not fully characterized (27).

*Clinical question (PIO) population*: Human patients with infected wounds; Intervention: silver ions released by commercially available dressings; Outcome: concentrations of silver ions released have a cytotoxic effect.

In view of these data, the research question (PIO) was: What concentrations of silver ions are released by dressings commercialized for the treatment of infected wounds? This led to the following clinical question: Is there a relationship between the concentrations of silver ions released by dressings in the treatment of infected wounds and cell toxicity?

The aim of this study was to evaluate the cytotoxic effects and the amount of silver released from commercially available dressings for patients with infected wounds.

## Methods

A systematic review of the literature was carried out according to the Preferred Reporting Items for Systematic Reviews and Meta-Analyses (PRISMA 2020) statement (28). This systematic review was registered in PROSPERO (CRD42022351041) (29).

### Eligibility criteria

The criteria were the following: population (human patients with infected wounds); intervention (commercial dressings with clinical silver authorized for use in humans); and outcomes (concentrations of silver ions released into tissues and plasma). The type of studies included were systematic reviews, experimental, quasi-experimental, and observational studies, with a sample size of more than five patients and published from 2002 through December 2022, in English, Spanish, or Portuguese.

Any study that failed to meet the aforementioned criteria, as well as those based on dressings without silver, experimental dressings, or dressings not yet clinically utilized in humans, were excluded.

### Search strategy

The search strategy was carried out in February 2023. The last search was done on 26 February. First of all, databases specialized in systematic reviews were consulted; no studies were found that met the defined criteria. Subsequently, a search for original studies was carried out in health databases (Web of Science, PubMed, Embase, Scopus, and CINAHL-Cumulative Index of Nursing and Allied Literature Complete). The search strategy used Boolean combinations of the following keywords: “silver”, “wounds and injuries,” “exudates and transudates,” “ions release,” and “toxicity” (Appendix 1). This phase was completed with a reverse hand search. Results were exported to Zotero v.6 reference manager to eliminate duplicates. Taking into account that managers do not detect 100% of duplicates, a manual review was carried out where titles, authors and year were compared.

### Selection of studies

**Figure 1:**
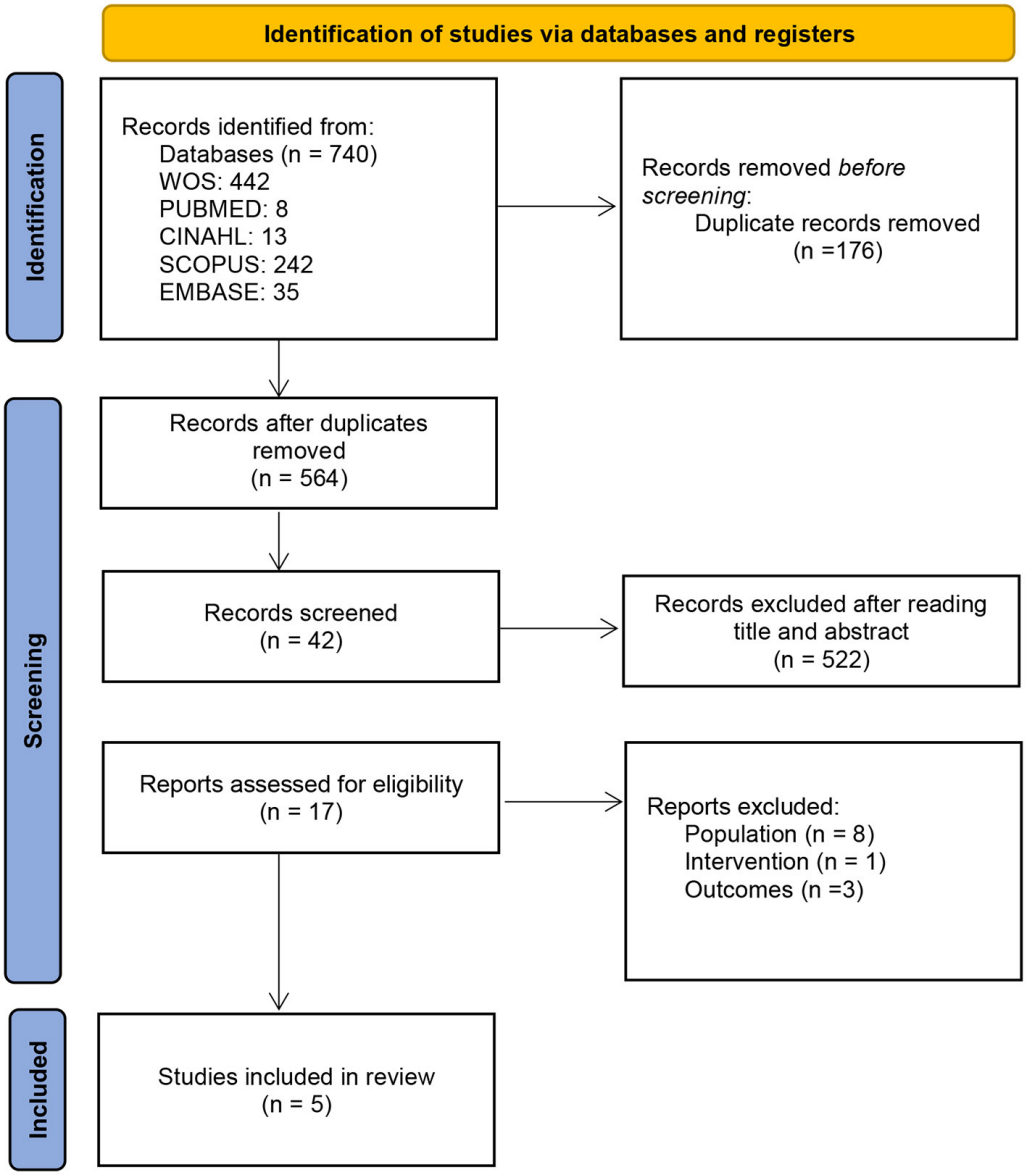
PRISMA flow diagram.

Two reviewers (JS and MH) independently selected the references according to the eligibility criteria. The selection was carried in 3 phases (title, abstract, and full text review). In case of disagreement, it was resolved by consensus between the two reviewers (Figure 1). In the case of disagreement, a third reviewer (SM) decided.

Following the PRISMA guidelines, the search strategy yielded a total of *n* = 740 articles of which *n* = 564 were assessed after removal of duplicates (*n* = 176). A first reading was performed by title and abstract to perform a first screening, eliminating those whose who did not met the expectations (*n* = 522). After, the articles that had not been eliminated in the previous phase were sought for retrieval and read (*n* = 17). Finally, each study was reviewed to choose those that met the study objectives. Twelve studies were ruled out, *n* = 8 for analyzing other population, *n* = 1 due to interventions that did not met the inclusion criteria and *n* = 3 that did not relate to the study outcomes. A total of *n* = 5 articles were finally selected (Figure 1).

### Evaluation of the quality

The reviewers adhered to the JBI Critical Appraisal Checklist for quasi-experimental studies, rigorously following the criteria outlined in the Explanation for the critical appraisal tool for quasi-experimental studies (30). The JBI critical appraisal tools were used for this purpose (31). Each study was independently evaluated by two reviewers (JS and MS), scoring the items with Yes, No, Unclear, Not applicable (Appendix 2). The reviewers were in complete agreement on 40% of the studies (2 out 5) and 89% agreement on the remaining 3 of the 5 articles included. Disagreements arose regarding JBI's item 3 in two studies, and item 8 in one study, both resolved by a third reviewer (SM). A value of 1 was assigned to each positive response, resulting in 60% of the accepted studies scoring 7 and 40% scoring 6 out of the 9 items analyzed. Studies that did not assess at least 65% of the items included (6/9) were considered to contain substantial methodological flaws.

### Analysis of variables and data extraction

Two reviewers independently selected the references according to the eligibility criteria and extracted data according a previously designed template: study (design and sample size); pathology (type of wound); intervention (type of silver dressing, follow-up quantification method); and effectiveness (concentration of silver released, cytotoxicity effects, and cytotoxicity rank). Data was processed and analyzed using the Microsoft Excel^®^ 2021 program.

### Data synthesis

The results were presented in the form of tables and charts, offering both total and percentage values, and as a narrative synthesis. Unfortunately, a meta-analysis could not be conducted due to the absence of a control group in the reviewed studies that met the inclusion criteria (involving commercial dressings and the determination of silver in humans with infected wounds). Control groups were identified in studies involving commercially available dressings *in vitro*, in some animal studies, and in those employing experimental dressings instead of commercial ones. While these studies could potentially facilitate a meta-analysis, they deviate from the practical application and outcomes in humans or with commercially available dressings. Additionally, these studies pose challenges for the interpretation of analyzed variables, as the methodology across different studies is not standardized.

### Ethical considerations

The systematic review was carried out using existing published data, with no identifiable information about individual patients. Consequently, approval from a research ethics committee was not deemed necessary.

## Results

The search for original articles yielded a total of 740 results. After screening and critical reading, five studies were selected (Figure 1).

### Study and sample size

All designs were quasi-experimental with no control group pre-post study and published between 2003 and 2014 (32–36). The sample size of the studies showed a variability from 6 (35, 36) to 46 (34), with the mean number of individuals per study being 22.6 (Table 1).

**Table 1:** Sociodemographic and wounds characteristics.

**Author (Year)**	**Patients (*n*)**	**Patients**			**Wound**		
		**Years**	**BMI**	**Sex**	**Size (cm** ^2^ **)**	**Location**	**Type**
		**Mean (minimum- maximum)**			**Mean (minimum- maximum)**		
Karlsmark et al. (32)	25	77 (59–93)	-	Male: 28%	15.6 (3.0–58.1)	Leg	Venous ulcers
Vlachou et al. (33)	30	38.9 (17–81)	26.4 (19.5–42.1)	Male: 83%	1486 (470–9862)	TBSA 7.3% (2.5–45%)	2nd-3rd degree burns 2–12% TBSA
Wang et al. (34)	46	3.9 (0 months-12 years)	-	-		TBSA 5% (0.5–40%)	2nd-3rd degree burns
Moiemen et al. (35)	6	37 (22–56)	-	Male: 83.3%	6166 (4718.1–13710.9)	TBSA: 31 (22–71%)	2nd-3rd degree burns 2–12% TBSA
Abarca-Buis et al. (36)	6	- (17–70)	-	Male: 50%	186.5 (15–450)		2 TW^*^, 2 SW^*^, 1 burn, 1 DF^*^

-, unknown; max-min, (minimum–maximum).

### Evaluation of the quality

Table 2 shows that the JBI Overall were in 60% (32–35), 7 about 9 (77.7%), and in 40% (35, 36), 6 about 9 (66.6%).

**Table 2:** Evaluation of the quality of the studies.

**Author (year)**	**Type of study**	**Reviewer**	**JBI critical checklist**									**JBI overall**		**Agreement**
			**1**	**2**	**3**	**4**	**5**	**6**	**7**	**8**	**9**			
Karlsmark et al. (32)	Quasi-experimental Pre-post	J	y	y	y	n	y	y	y	y	u	7	7	100%
		M	y	y	y	n	y	y	y	y	u	7		
Vlachou et al. (33)	Quasi-experimental	J	y	y	**n**	n	y	y	y	y	y	7	7	89%
		M	y	y	**n**	n	y	y	y	y	y	7		
Wang et al. (34)	Quasi-experimental	J	y	y	n	n	y	y	y	y	y	7	7	100%
		M	y	y	u	n	y	y	y	y	y	7		
Moiemen et al. (35)	Quasi-experimental	J	y	y	y	n	y	y	y	**n**	n	6	6	89%
		M	y	y	y	n	y	y	y	**n**	n	6		
Abarca-Buis et al. (36)	Quasi-experimental	J	y	y	**y**	n	y	y	y	n	n	6	6	89%
		M	y	y	**y**	u	y	y	y	n	n	6		

y, Yes; n, No; u, Unclear; na, Not applicable; In bold: disagreement.

With respect to the JBI tools items (Appendix 2), it was observed that items 1, 2, 5, 6, and 7 were achieved by all the articles and Item 4, none of them reached because had no control group.

Abarca-Buis et al. (36) and Moiemen et al. (35), did not measure the outcomes reliably (item 8) and did not use the appropriate statistics (item 9). The same was observed in Karlsmark et al. (32), they did not use the appropriate statistics (item 9).

### Socio-demographic characteristics of patients

In 4 studies (32–35) the patients were adults aged 17–93 years and Wang et al. (34) were children. Gender was shown in 4 of the 5 articles (32–35) with percentages of men between 28.8 and 83.3 and Body mass index in Moiemen et al. (35) (Table 2).

### Characteristics of wounds

Regarding wound characteristics, wound size was shown in 4 of the 5 studies with ranges from 15.6 cm^2^ to 6166 cm^2^. Regarding location, the Karlsmark et al. (32) article located the wounds on the leg and 3 of the articles (33–35) that the wound type was burns, provided the TBSA (Table 2).

The types of injuries were burns (69.2%) (33–35), venous vascular ulcers (26.7%) (32), surgical wounds (1.7%), traumatic wounds (1.7%), and diabetic foot (0.8%) (36). The variability in the etiology of treated wounds, although not directly related to silver release, makes it difficult to standardize results.

### Pathology

The article published by Moiemen et al. (35) reported that 50% of the patients were smokers and Karlsmark et al. (32), 8% were non-insulin dependent diabetics. The other articles did not report patient health data.

Regarding the type of dressing (Table 3), nanocrystalline (60%), ionic (20%), and metallic (20%) silver dressings were used.

**Table 3:** Silver release.

**Author (Year)**	**Type of wound**	**Silver release (Blood serum) (minimum- maximum)**	**Silver release (Wound exudate)**	**Type of silver dressing**	**Follow-up**	Quantification method^**^
Karlsmark et al. (32)	Venous ulcers	4 nmol/l−80 nmol/l (0.0004 ppm−0.0086 ppm)	NA	Ionic silver	28 days	AAS
Vlachou et al. (33)	2nd-3rd degree burns	1.1 y 230 μg/L (0.0011 ppm−0.230 ppm)	NA	Nanocrystalline	9 days	ICPMS
Wang et al. (34)	2nd-3rd degree burns	5.4 μg/L −735 μg/L (0.0054 ppm−0.735 ppm)	NA	Nanocrystalline	18.5 days	ICPMS
Moiemen et al. (35)	2nd-3rd degree burns	50 μg/L −483 μg/L (0.005 ppm−0.483 ppm)	NA	Nanocrystalline	9 days	ICPMS
Abarca-Buis et al. (36)	2 TW^*^, 2 SW^*^, 1 burn, 1 DF^*^	< 14.2 μg/L (0.0142 ppm)	< 90100 μg/L (90.1 ppm)	Metallic	3 days	GFASS

^*^TW, Traumatic wound; SW, Surgical wound; DF, Diabetic Foot; ^**^AAS, Atomic Absorption Spectroscopy; ICPMS, Inductively Coupled Plasma Mass Spectrometry; GFAAS, Graphite Furnace Atomic Absorption Spectrometry.

The assessment of the concentrations of silver released was performed in all studies using a blood serum sample, giving values between 0.0011 ppm and 0.735 ppm (parts per million) for nanocrystalline silver dressings (33–35), below 0.0142 ppm for metallic silver (36), and 0.0004 ppm−0.0086 ppm for ionic silver (32). The quantification method used was inductively coupled plasma mass spectrometry (ICPMS) in 60% of the cases (33–35), graphite furnace atomic absorption spectrometry (GFAAS) in 20% (36), and atomic absorption spectroscopy (AAS) in 20% (32). The mean dressing wear time was 13.5 days (with variability between 3 and 28 days) (32, 36).

Regarding cytotoxicity (Table 4), no study presented data on cytotoxicity beyond indicating the absence of clinical symptoms. Only Karlsmark reported silver deposits in the liver and kidney, but both maintained normal function, at least during the follow-up time (32).

**Table 4:** Cytotoxicity.

**Study**	**Cytotoxicity**	**Cytotoxic range in blood (>0.01 ppm)**	**Consequences**
Karlsmark et al. (32)	No	No	Deposits in liver and kidney
			Normal function
Vlachou et al. (33)	No	Yes	No clinical symptoms
Wang et al. (34)	No	Yes	No clinical symptoms
Moiemen et al. (35)	No	Yes	No clinical symptoms
Abarca-Buis et al. (36)	No	Yes	No clinical symptoms

## Discussion

The aim of this study was to assess both the cytotoxicity and the quantity of silver released by commercially accessible dressings.

The number of identified studies was small (a total of five). No control group was used in all the studies and in 3 (32, 35, 36) of them descriptive statistics were performed only on the results found. This selection of study design has as a consequence the possibility of possible biases.

Although the number of articles in our review was small, studies on *in vitro* silver release or those using experimental dressings were numerous. One of the difficulties encountered when analyzing studies on silver released by commercially available dressings was the sampling and quantification of the silver released, hence the small number of final articles. In these 5 included studies, the results of 113 patients were analyzed, ranging from 6 (35, 36) to 46 (34) patients depending on the study.

In terms of wound description and characteristics, problems were observed in determining the size of the wounds. Only Wang et al. (34) indicated that the size of the treated wound was related to higher blood silver concentrations.

The amount of exudate identified when dealing with the wound was not specified. The release of silver from the dressing in the form of ions is dependent on moisture, so there is a direct relationship between the amount of exudate in the wound and the amount of silver released into the wound. This amount of exudate is also related to the type of tissue on which the dressing is applied, although this is not described in the literature (37).

Other relevant aspects in the assessment of the amount of silver released were continued application and the frequency of dressing change. It was noted that this was not reflected in the studies (38) and therefore could not be compared. Silver-releasing dressings can be effective for up to 7 days, depending on the type of dressing, although the wound is usually dressed every 48 h−72 h, depending on the wound exudate (4). These variables should be included in future studies.

*In vivo* comparative studies of clinical dressings for control of the infection lack a standardized methodology that allows observation of all the variables of silver performance at local and systemic levels (39).

The quantification methods used varied across studies, with ICPMS, GFAAS, and AAS being identified. The main difference between these quantification methods is the detection limit, which is 0.00001 ppm−0.0001 ppm for ICPMS, 0.00005 ppm for GFAAS, and 0.002 ppm for AAS (40). When analyzing the studies, values below the bactericidal range were observed for released silver, which is considered to be bactericidal at 1 ppm and above (41), despite the fact that blood measurements, which are useful for determining cytotoxicity, are not directly related to the values of silver present in the wound bed, where the bacterial load is located. Only Abarca-Buis et al. provided data on silver concentrations in exudates (36), obtaining a bactericidal value of 90.1 ppm, as the effective inhibitory concentrations of silver nanoparticles varies between 10 ppm and 100 ppm (22). Exudate or wound biopsy measurements could yield a more accurate result on the concentration of silver released into the wound bed. There are therefore differences depending on the type of silver used, the particle size, and the way it is bound to the different layers of the dressing, as well as the type of dressing used (9).

A better understanding of the wound microenvironment is essential for the creation of innovative wound dressings specifically crafted to enhance the healing process (42).

There is currently great heterogeneity among recommendations and clinical practice guidelines as to how to apply dressings to control or reduce wound bacterial load (43). Across the different analyzed articles, differences in criteria and no standardization can be observed with regard to dressing type, application protocol, detection method, and *in vivo* results (32–36). Upon comparison of these results with those obtained in *in vitro* studies, similar difficulties to those *in vivo* were observed with respect to the methodology applied, measurement times, and quantification method, as well as the type of solution and prior saturation of the dressing (13, 44, 45).

No study provided specific data on cytotoxicity nor did any indicate the cytotoxicity levels for silver. In any case, 80% exceeded the range of silver in blood that, to date, is considered cytotoxic (>0.01 ppm) (19). The study by Karlsmark et al. (32) was the only one outside the cytotoxic range in blood, but its results indicated hepatic and biliary deposits. We conducted an extensive literature review focusing on the updated cytotoxicity threshold for silver. Reported instances of localized argyria have been associated with exposure to silver ions, metallic surfaces, and nanocrystalline silver. Generalized argyria occurred in humans exposed to ionic and nanocrystalline silver at cumulative doses ranging from 70 mg to 1500 mg silver/kg body weight. Despite a low potential for skin irritation, eye irritation, and occasional instances of allergic contact dermatitis, additional data are required to assess the genotoxic and carcinogenic potential of silver (13).

The genotoxic potential of all metal or metal oxide nanoparticles *in vitro* and/or *in vivo* has been suggested. However, inconsistent results have been observed (46). This characterization is indispensable for reliable toxicity studies (47). Cytotoxicity linked to silver nanoparticle size was observed, with smaller sizes exhibiting higher toxicity. Dose-dependent toxicity was noted, with higher doses correlating to increased toxicity. The nanoparticle's form also played a role, with spherical nanoparticles showing heightened toxicity compared to other forms (48). The precipitation of silver, and its association with protein complexes, favors its penetration (44). Despite the ability of silver ions and other metals to penetrate and permeate skin layers, no reliable studies have been carried out that allow a full understanding of their transfer *in vivo* (49). The toxicity of silver nanoparticles affects all cell types, regardless of their level of complexity, although manufacturers of silver-releasing dressings have not described penetration values (22). The physical and chemical characterization of nanoparticles is a preliminary and essential step to carry out reliable studies that provide insight into their toxicity (47). The absence of penetration capacity values for skincare products containing potentially toxic metals is attributed to incomplete particle characterization, raising ethical concerns about producing and supplying such products without a clear understanding of their toxicity and long-term effects (27). Obtaining specific and definitive data on the physico-chemical properties, toxicological profiles in mammalian systems, environmental fate, and risk assessment of materials releasing silver ions should be the basis prior to their application, despite the apparent absence of cytotoxic effects derived from their use.

The main limitations encountered in carrying out this study were the absence of a control group in all the studies and the heterogeneity found in the sampling. In turn, the studies analyzed during the review process offered results in different units of measurement (mg/cm^2^, μg/g, μg/kg, μg/L, μg/ml, nmol/l…); standardizing them to the same research unit (ppm) would facilitate comparison between studies. The data analyzed did not provide cytotoxic ranges to make an assessment or even a comparison between studies.

The limitations have been addressed, and the present study highlights a primary risk, namely selection bias. The analyzed studies and the applied methodology, comprising quasi-experimental studies without a control group and randomization, contribute to studies with a high risk of this type of bias.

Despite conducting a thorough and systematic search, there is a potential for publication bias. Negative results may not have been published and/or may be unavailable in the scientific literature, leading to an overestimation of the intervention effects.

Confounding bias may be present in our review. The absence of inferential statistics or the analysis of health factors as confounding variables may render the results susceptible to uncontrolled influences, thereby complicating the accurate interpretation of the causal relationship between variables.

Other biases that may have influenced our results include detection bias, given the absence of a standardized method for measuring silver in blood, and sample size bias, which was not calculated in any of the studies.

The clinical implications of this study highlight the lack of homogeneity in the application of silver-releasing dressings to the wound bed, along with the limited understanding of the cytotoxic profile of commercially available silver-containing dressings. The manner in which the dressing is (or is not) released, as well as the type of dressing used, alters the application approach, dressing change intervals, and the overall duration of silver treatment on the wound bed.

Our study revealed differences in the application of silver-containing products concerning the type of wound rather than the specific products used. Nanocrystalline silver dressings, such as those employed by Vlachou et al. (33), Wang et al. (34), and Moiement et al. (35), involve a high release of silver to the wound bed, effectively controlling bacterial load within 48 hours. These dressings require a standardized pre-wetting with sterile water and the maintenance of moisture in the wound bed between dressings. Dressings containing metallic silver, as utilized by Abarca-Buis et al. (36), also exhibit sustained silver release to the wound bed and do not require pre-wetting. Differentiating between a polyamide mesh impregnated with fatty acid ointment (50), a hydroalginate with a non-adherent layer (51), or a hydrophobic polyurethane silver loaded foam is crucial to avoid adherence or damage to the wound bed (52). The exudate management capacity and saturation of these products in the presence of exudate vary significantly. The removal of hydroalginate dressings may necessitate the application of moisture for less exudative wounds. The last type of silver analyzed in reviewed studies is ionic silver, specifically linked to a soft hydrophilic polyurethane foam containing silver as an integral part of the matrix (32). This dressing is recommended for use on moderately to highly exuding wounds with bacterial burden, releasing silver ions in the presence of wound exudate (53).

Therefore, the lack of homogeneity emphasizes the necessity for standardized protocols in the application of silver-releasing dressings. These protocols should differentiate the mode of action among various dressings and include the quantification of the amount of silver released over time.

Despite evidence supporting the use of silver-containing therapeutic clothing without side effects in patients with atopic dermatitis (54), the prolonged use of silver-releasing dressings raises concerns regarding the proposed risk of systemic toxicity due to improper usage. The potential for localized skin pigmentation is elevated, especially with nanocrystalline silver dressings, though studies clarifying the issues associated with extended use are lacking. Prolonged use is discouraged, similar to any other antimicrobial agent. If a wound does not show a positive response to silver dressings within 14 days, a change in treatment is considered, often involving systemic antibiotic therapy. Proper application of different dressings to enhance their bactericidal action is crucial to ensure the appropriate release of silver.

Perhaps the key lies in conducting *in vitro* experiments in a translational manner, mirroring how the dressing would be applied in clinical practice. In the case of clinical trials, it is crucial to conduct them with a control group and adhere to consistent dressing change schedules, as well as to employ the same method for silver determination. While systemic toxicity from silver is rare, the minimum amount required to induce it remains unknown. Conducting studies to understand the penetration capacity through healthy skin and wound bed, as well as determining systemic toxicity levels of silver, would enable the development of an accurate toxicological profile before its use in wound healing dressings and other products in direct contact with the skin.

The correlation between the actual amount of silver released by dressings into the wound bed and its absorption has yet to be established. Exploring this research avenue would contribute to preventing potential future toxicities, providing valuable insights into the dynamics of silver absorption from wound dressings.

## Conclusion

This review highlights the methodological variability in experimentation in this field. This variability hinders comparison across studies and dressings, so a future approach would be to standardize the experimental methodology for the study of silver release from wound healing dressings, making this a more translational field to better understand what actually happens in a wound.

The assessed studies showed that the quantification of silver release was below the bactericidal range and did not take into account aspects that may be determinants such as exudate, dressing wear time, and frequency of change. This quantification in translational *in vitro*/*in vivo* research would provide a clear timeframe-related range. This could enhance the clinical use of silver-releasing dressings for wounds while also preventing entry into the cytotoxic range. Based on the obtained results, it cannot be concluded whether the assessed concentrations of silver released by commercially available dressings for the topical treatment of infected wounds is cytotoxic to skin cells.

## Data availability statement

The original contributions presented in the study are included in the article/supplementary material, further inquiries can be directed to the corresponding author.

## Author contributions

JS-G: Conceptualization, Eligibility criteria, Investigation, Methodology, Project administration, Data synthesis, Supervision, Visualization, Writing—original draft, Writing—review & editing. SM-I: Conceptualization, Eligibility criteria, Investigation, Methodology, Project administration, Data synthesis Supervision, Visualization, Writing—original draft, Writing—review & editing. JG-S: Conceptualization, Data synthesis, Visualization, Writing—original draft, Writing—review & editing. JR-P: Conceptualization, Data curation, Formal analysis, Investigation, Methodology, Resources, Software, Supervision, Validation, Visualization, Writing—original draft, Writing—review & editing. MS-P: Conceptualization, Bibliographic search, Eligibility criteria, Supervision, Validation, Visualization, Writing—original draft, Writing—review & editing. MS-H: Conceptualization, Eligibility criteria, Data synthesis, Methodology, Resources, Software, Supervision, Validation, Visualization, Writing—original draft, Writing—review & editing. MG-M: Conceptualization, Data curation, Formal analysis, Investigation, Methodology, Resources, Software, Supervision, Validation, Visualization, Writing—original draft, Writing—review & editing. DF-G: Data curation, Formal analysis, Investigation, Methodology, Project administration, Resources, Software, Supervision, Validation, Visualization, Writing—review & editing.

## Appendix 1. Search strategy

**Table:**

**Database**	**Search strategy**
**PUBMED**	(“Bandages”[Mesh] OR dressing^*^ OR bandage^*^) AND (silver^*^ OR “silver”[MeSH Terms] OR “silver compounds”[MeSH Terms]) AND (wound^*^ OR “Wounds and Injuries”[MeSH Terms] OR “injuries”[MeSH Subheading]) AND (“exudates and transudates”[MeSH Terms] OR Infect^*^ OR Contamin^*^) AND (“release experiments” OR ion-exchang^*^ OR ion-liberation^*^ OR concentrate^*^ OR “Silver Release” OR “Ions release” OR “Ions exchange” OR “Ions concentration” OR toxicit^*^ OR cytotoxicit^*^ OR biocompatibilit^*^ OR Absorpti^*^) Limits: Systematic review, clinical trial, or observational study. English, Spanish, or Portuguese. Humans. 2002–2022 Outcomes: 8 results
**WOS**	(dressing^*^ OR “bandages”) AND (silver^*^) AND (wound^*^ OR injur^*^) AND (exudat^*^ OR transudate^*^ OR Infect^*^ OR Contamin^*^) AND (“release experiments” OR “ion-exchange” OR “ion-liberation” OR concentrat^*^ OR Release^*^ OR “Ions exchange” OR toxicit^*^ OR cytotoxicit^*^ OR biocompatibilit^*^ OR Absorpti^*^) Limits: Article. English, Spanish, or Portuguese. Humans. 2002–2022 Outcomes: 442 results
**EMBASE**	(dressing^*^ OR “bandages”) .ab AND (silver^*^) .ab AND (wound^*^ OR injur^*^) .ab AND (exudat^*^ OR transudate^*^ OR Infect^*^ OR Contamin^*^) .ab AND (“release experiments” OR “ion-exchange” OR “ion-liberation” OR “silver concentration” OR Release^*^ OR “Ions exchange” OR OR toxicit^*^ OR cytotoxicit^*^ OR biocompatibilit^*^ OR Absorpti^**^).ab Limits: Article. English, Spanish, or Portuguese. Humans. 2002–2022 Outcomes: 35 results
**CINAHL**	(dressing^*^ OR “bandages”) AND (silver^*^) AND (wound^*^ OR injur^*^) AND (exudat^*^ OR transudate^*^ OR Infect^*^ OR Contamin^*^) AND (“release experiments” OR “ion-exchange” OR “ion-liberation” OR concentrat“ OR Release^*^ OR “Ions exchange” OR toxicit^*^ OR cytotoxicit^*^ OR biocompatibility^*^ OR Absorption^*^) Limits: Article. English, Spanish, or Portuguese. Humans. 2002–2022 Outcomes: 13 results
**SCOPUS**	(dressing^*^ OR bandages^*^) AND (silver^*^) AND (wound^*^ OR injur^*^) AND (exudat^*^ OR transudate^*^ OR Infect^*^ OR Contamin^*^) AND (“release experiments” OR “ion-exchange” OR “ion-liberation” OR concentrat^*^ OR Release^*^ OR “Ions exchange” OR toxicit^*^ OR cytotoxicit^*^ OR biocompatibilit^*^ OR Absorpti^*^) Limits: Article. English, Spanish, or Portuguese. Humans. 2002–2022 Outcomes: 242 results

## Appendix 2. JBI critical appraisal checklist

**Table:**

1	Is it clear in the study what is the ‘cause' and what is the ‘effect' (i.e. there is no confusion about which variable comes first)?
2	Were the participants included in any comparisons similar?
3	Were the participants included in any comparisons receiving similar treatment/care, other than the exposure or intervention of interest?
4	Was there a control group?
5	Were there multiple measurements of the outcome both pre and post the intervention/exposure?
6	Was follow up complete and if not, were differences between groups in terms of their follow up adequately described and analyzed?
7	Were the outcomes of participants included in any comparisons measured in the same way?
8	Were outcomes measured in a reliable way?
9	Was appropriate statistical analysis used?
